# Dynamic network biomarker identifies *cdkn1a*-mediated bone mineralization in the triggering phase of osteoporosis

**DOI:** 10.1038/s12276-022-00915-9

**Published:** 2023-01-04

**Authors:** Weiming Guo, Peng Jin, Ruomei Li, Lu Huang, Zhen Liu, Hairui Li, Ting Zhou, Bing Fang, Lunguo Xia

**Affiliations:** 1 Department of Orthodontics, Shanghai Ninth People’s Hospital, Shanghai Jiao Tong University School of Medicine; College of Stomatology, Shanghai Jiao Tong University; National Center for Stomatology; National Clinical Research Center for Oral Diseases; Shanghai Key Laboratory of Stomatology, Shanghai, 200001 China; 2 Shanghai Institute of Hematology, State Key Laboratory of Medical Genomics, National Research Center for Translational Medicine at Shanghai, Ruijin Hospital, Shanghai Jiao Tong University School of Medicine, Shanghai, 200001 China; 3 Department of Plastic and Reconstructive Surgery, Shanghai Ninth People’s Hospital, Shanghai Jiao Tong University School of Medicine, Shanghai, 200001 China

**Keywords:** Gene ontology, Predictive markers

## Abstract

The identification of predictive markers to determine the triggering phase prior to the onset of osteoporosis is essential to mitigate further irrevocable deterioration. To determine the early warning signs before osteoporosis, we used the dynamic network biomarker (DNB) approach to analyze time-series gene expression data in a zebrafish osteoporosis model, which revealed that cyclin-dependent kinase inhibitor 1 A (*cdkn1a*) is a core DNB. We found that *cdkn1a* negatively regulates osteogenesis, as evidenced by loss-of-function and gain-of-function studies. Specifically, CRISPR/Cas9-mediated *cdkn1a* knockout in zebrafish significantly altered skeletal development and increased bone mineralization, whereas inducible *cdkn1a* expression significantly contributed to osteoclast differentiation. We also found several mechanistic clues that *cdkn1a* participates in osteoclast differentiation by regulating its upstream signaling cascades. To summarize, in this study, we provided new insights into the dynamic nature of osteoporosis and identified *cdkn1a* as an early-warning signal of osteoporosis onset.

## Osteoporosis: finding the earliest signs

New techniques that allow researchers to visualize gene network activation in time-lapse have revealed a key gene implicated in the earliest phase of osteoporosis. Osteoporosis affects millions worldwide, but most cases are only diagnosed after a bone has been broken and significant bone loss has already occurred. A method for detecting the pre-disease phase could help in preventing severe disease. Weiming Guo at Shanghai Jiao Tong University, China, and co-workers mapped gene activation over time to detect the genes involved in the very first stages of osteoporosis. They identified a key gene, *cdkn1α*, and determined that it suppresses bone formation when osteoporosis begins. Deleting the gene in zebrafish, a model organism for studying osteoporosis, improved bone mineralization. These results may help both in identifying patients with earliest-stage osteoporosis and in developing better treatments.

## Introduction

Osteoporosis, characterized by decreased bone mass, increases the risk of bone fracture, thereby substantially decreasing quality of life^1^. Osteoporosis can be divided into primary and secondary osteoporosis. Primary osteoporosis may result from aging or estrogen deficiency, and secondary osteoporosis may be caused by chronic treatment with glucocorticoids or other factors^2–4^. According to the World Health Organization, in 2000, an estimated 9.0 million (0.18%) individuals suffered from osteoporotic fractures globally^5^. Furthermore, many osteoporosis patients are not diagnosed until the occurrence of bone fractures^3^. It was reported that three-quarters of women experiencing a fracture did not meet the current clinically used guidelines for osteoporosis screening^6^. Accordingly, a greater understanding of the pathogenesis and molecular mechanisms that contribute to irrevocable osteoporosis remains a high priority, not only for preventing the initiation of osteoporosis from the predisease state but also for developing therapeutic approaches to treat osteoporosis patients.

The development and formation of osteoporosis is a dynamic process. Although several potential genes and proteins can be applied in the diagnosis of osteoporosis, it is challenging to establish the triggering phase prior to osteoporosis initiation for early diagnosis and intervention. The nondisease (normal) phase and the predisease stage are remarkably different dynamically, so it should be possible to establish dynamic markers (instead of traditional static markers) for forecasting the predisease phase of osteoporosis^7^. Herein, we employed a mathematical approach, namely, the dynamic network biomarker (DNB) model, to determine the predisease phase or triggering phase by assessing the dynamic omics information from animal models^8^. Compared with traditional biomarkers, which detect the disease state based on the differential expression of molecules, a major advantage of the DNB method is its ability to identify the triggering phase in disease progression.

Although murine animal models of osteoporosis have been used for many years, they have numerous disadvantages, such as long lead times, high costs, and high labor intensity. However, zebrafishes have recently been identified as an optimal model for observing skeletal development because of their small size, transparency, easy rearing, and short generation time. Numerous studies have shown that the hormonal response of zebrafish to glucocorticoids is similar to that of humans, which provides an attractive nongenetic model for bone formation and mineralization^9,10^. In previous research, the larval zebrafish model of glucocorticoid-induced osteoporosis has been widely used as a proven model^11,12^. To directly determine and quantify the dynamics of osteoporosis, we specifically adopted a transgenic zebrafish called the Tg (*Ola. Sp7: nlsGFP*) zebrafish. Its bone tissue expresses green fluorescent protein (GFP) under the control of the osterix (*sp7*) promoter, which is a preosteoblast intermediate marker^12,13^. By analyzing the time-series whole-genome expression profiles of zebrafish osteoporosis samples based on the DNB approach, we identified drastic changes on the 6th day after the addition of dexamethasone (DEX), which corresponded to the predisease state. Furthermore, we discovered several DNB members that play a precursor role in initiating osteoporosis in zebrafish models and, hence, can be used as predictive biomarkers for osteoporosis.

In this study, we established the DNB approach to estimate the critical transition in osteoporosis, and we provide novel insights into the molecular pathology of osteoporosis on the basis of dynamics and networks. Of note, we identified cyclin-dependent kinase inhibitor 1 A (*cdkn1a*) as a dynamic signature. The CRISPR/Cas9 gene editing technique (where CRISPR denotes clustered regularly interspaced short palindromic repeats and Cas9 denotes CRISPR-associated protein 9) was used to further elucidate the role of *cdkn1a* in zebrafish osteoporosis, and the results identified *cdkn1a* as an early-signaling indicator of the onset of osteoporosis.

## Materials and methods

### Zebrafish husbandry

We reared and maintained wild-type (WT) and Tg (*Ola.Sp7: nlsGFP*) zebrafish following standard protocols. Transgenic lines were reported previously^14^. We reared adult zebrafish under recirculating water conditions at 14/10 h light/dark (L/D) phases at 28 °C. We fed the zebrafish three times a day. To generate embryos, we paired the male zebrafish with females in the evening, with spawning occurring the following day within 1 h after turning the lights on.

### Establishment of the osteoporosis model

The basis for the rapid induction of osteoporosis by glucocorticoids in zebrafish lies in the fact that ossification of the craniofacial skeleton in zebrafish starts at ~3 days post-fertilization (dpf)^14–19^, and the cephalic bones develop between 3 and 9 dpf^13,20^. Several studies have found that osteoporosis in zebrafish larvae induced by glucocorticoids can be evident within 5 days^10,12,13,16,21,22^. Based on the above evidence, in our study, we exposed the Tg (*Ola.Sp7: nlsGFP*) transgenic zebrafish larvae to DEX continuously for 5 days from Day 3 to Day 7, and we inoculated the sibling larvae with dimethyl sulfoxide (DMSO) to serve as the controls. The concentration of DEX used in the experiment was 10 µM, which is nontoxic and effective, as reported in several previous studies^11,13,22–24^. We reared all zebrafish larvae in culture dishes and changed the treatment solutions daily until the sampling time.

### Whole-mount skeletal staining

First, the larvae were fixed in 2% PFA overnight and then rinsed in egg water. Next, the larvae were stained overnight in 0.5% Alizarin red stain solution and then bleached with 1.5% hydrogen peroxide/1% KOH at room temperature for 20 min, followed by rinsing overnight twice with 50% glycerol/0.25% KOH. We employed a stereomicroscope (Nikon, SMZ25, Japan) to acquire images. Finally, we analyzed the digital images for quantification and comparison of the stained area and staining density.

### Imaging and evaluation of bone mineralization

We employed a stereomicroscope (Nikon, SMZ25, Japan) to image the Tg (*Ola.Sp7: nlsGFP*) transgenic zebrafish. Similar parameters were used in the process of imaging all zebrafish from the same set of experiments. For transgenic fish, we first inverted the fluorescent images to grayscale images. Afterward, we determined the fluorescent area and integrated optical density (IOD), which reflected the degree of mineralization and bone mineral density. All image processing was performed using ImageJ software.

### RNA-sequencing (RNA-seq) and analysis

We collected samples treated with DEX, as well as those from WT and cdkn1a^Cas9/sgRNA^ crispants, at 3, 4, 5, 6, and 7 dpf to perform RNA-seq. Samples were from whole larvae. Total RNA was extracted and purified with TRIzol (Invitrogen, Carlsbad, CA, USA) as described by the manufacturer. RNA quantity and purity were determined with a NanoDrop ND-1000 (NanoDrop, Wilmington, DE, USA) and Bioanalyzer 2100 (Agilent, Santa Clara, CA, USA). Only RNAs with a high RNA integrity number (RIN > 7) were selected and used for subsequent sequencing. Sequencing (150-bp paired-end reads) was performed on the Illumina Novaseq™ 6000 (LC-Bio Technology Co., Ltd., Hangzhou, China). Trim-Galore was utilized to remove the adapter sequences, and then, the clean reads were aligned with STAR (v2.7.8) and quantified with HT-Seq (v0.13.5) using the GRCz11 zebrafish assembly. The differential expression assessment was conducted using the DESeq2 package. DEGs used for unsupervised hierarchical clustering were generated using five two-class comparisons (D3 vs. other, D4 vs. other, D5 vs. other, D6 vs. other, and D7 vs. other). Gene Ontology (GO) and Kyoto Encyclopedia of Genes and Genomes (KEGG) enrichment analyses were carried out using Metascape. Gene set enrichment analysis (GSEA) and gene set variation analysis (GSVA) were performed using clusterProfiler and GSVA packages, respectively. The total genes of “osteoblast differentiation” and “osteoclast differentiation” are shown in Supplementary Table 5. Based on the osteoblastic/osteoclastic differentiation pathway signatures obtained from the GO, we first scored all the zebrafish samples using GSVA. Next, the GSVA scores of zebrafish samples at the indicated timepoints (D3 to D7) were visualized. The microarray expression data of mesenchymal stem cell populations in primary osteoporosis and healthy donors were obtained from GSE35959, and differential expression was determined using the limma package. The RNA-seq data generated in this study have been deposited at Gene Expression Omnibus (GEO) under accession code GSE194301.

### DNB analysis

The development of osteoporosis can be modeled by three stages: (1) the nonosteoporosis phase with high robustness and resilience to perturbations; (2) the preosteoporosis phase, which is typified by low robustness and resilience given its critical dynamics but is still revertible to the nonosteoporosis phase with specified treatments; (3) and the osteoporosis condition, which is another stable state with high robustness and resilience and is thus a challenge to revert to the nonosteoporosis condition even with advanced treatments. On the basis of nonlinear dynamic theory, when the system is approaching the triggering phase, there is a predominant group of genes consisting of DNBs satisfying the following three criteria:

(1) Standard deviations (*SD*_*in*_) of genes in this group are dramatically high;

(2) The Pearson correlation coefficients (*PCC*_*in*_) of genes in this group are remarkably higher than those of others;

(3) Pearson correlation coefficients between genes in this group and others (*PCC*_*out*_) are markedly reduced.

The quantification metric (*CI*: criticality index) considers all three criteria and is widely utilized as the numerical signal in the DNB approach:

$$CI = \sqrt {size} \frac{{PCC_{in}}}{{PCC_{out}}}SD_{in}$$

where *size* is the number of genes, *SD*_*in*_ is the average standard deviation of all genes in the DNB group, *PCC*_*in*_ is the average PCC of all gene pairs in the DNB group (absolute value), and *PCC*_*out*_ is the average PCC of molecule pairs between the dominant group and others (absolute value).

### Ranking scheme for DNB members

We comprehensively ranked the DNB genes according to the integrated importance index considering two priorities. First, we ranked DNB genes for their significance at the network level. Briefly, we mapped the DNB genes into a molecular network and calculated the network degree of each DNB gene. Then, we normalized the degree of each DNB gene to the total number of links among all DNB genes (index one). Next, we ranked DNB genes according to their roles at the biological cascade level. We counted the number of KEGG cascades in which each DNB gene was involved, and we then ranked the DNB genes in descending order. In this study, we considered 185 pathways obtained from the Molecular Signatures Database. We also normalized the pathway degree of each DNB gene to 185 and represented it as a percentage (index two). Finally, we generated an integrated index combining index one and index two for comprehensively ranking the DNB genes.

### sgRNA design, synthesis and microinjections

All single guide RNAs (sgRNAs) were selected from the priority targets determined by CHOPCHOP software (http://chopchop.cbu.uib.no/) and were designed based on the beginning of exon 2 of *cdkn1a* (Supplementary Table 1 and 2)^25^. PCR with the pYSY-gRNA vector (YSY, China) was used to amplify the sgRNA templates, and then the MAXIscript In Vitro Transcription kit (Ambion, Austin, TX, USA) was used to synthesize sgRNAs^26^. Four chosen pooled sgRNAs were mixed with Cas9 protein reconstituted in nuclease-free water, incubated at 37 °C for 5 min, and then microinjected into the one-cell stage of zebrafish embryos. The final injection amounts for each embryo were 400 ng of Cas9 protein and 250 ng of sgRNA pool.

### mRNA synthesis and microinjections

To synthesize mRNA, we cloned the full-length coding sequence of *cdkn1a* by RT‒PCR based on cDNAs derived from zebrafish embryos. We then subcloned the PCR products into the pXT-7 vector in the direction of the T7 promoter^26^. The mMESSAGE mMACHINE T7 Ultra Transcription Kit (Ambion, Austin, TX, USA) was used to synthesize cap and tail *cdkn1a* mRNAs in vitro, followed by microinjection into the one-cell stage of zebrafish embryos. The final injection amount for each embryo was 50 pg of *cdkn1a* mRNA.

### Identification and screening of *cdkn1a*-mutant zebrafish

To establish a pure knockout line, we collected fertilized eggs and performed microinjections when the embryos were in the single-cell stage, thoroughly mixing Cas9 mRNA and *cdkn1a*-active sgRNA before injection (Supplementary Fig. 4a). Then, we selected the same batch of injected F0 embryos with a high knockout efficiency and survival rate to grow to sexual maturity and identified the genotype by sequencing. We mated the screened positive F0 zebrafish with WT zebrafish and then fed the F1 embryos as usual until they were more than 2 months old for genotyping to obtain the knockout mutant F1 generation. We inbred F1 zebrafish carrying the same mutation to obtain the F2 generation, and genotype identification of the F2 generation screened out *cdkn1a* homozygotes. The screening process is shown in Supplementary Fig. 4b.

### Real-time PCR

We employed the RNAiso Plus reagent (TaKaRa, 9108, Japan) to isolate total RNA, and we generated cDNA from the RNA with the PrimeScript RT reagent Kit (TaKaRa, RR420A, Japan). Next, we utilized the SYBR Green-based real-time detection approach to perform q-PCR on the ABI system. We employed the 2^−ΔΔCt^ approach to determine relative mRNA expression. Beta-actin served as the normalization gene. The PCR oligonucleotide primers are listed in Supplementary Table 3.

### Statistical analysis

We replicated all experiments at least three times and reported the data as the mean ± SD. Statistical analysis was performed with GraphPad Prism V.8 software (San Diego, CA, USA). Differences between the two groups were tested with Student’s *t*-test. Multiple groups were compared using one-way ANOVA. *P* < 0.05 indicated statistical significance.

## Results

### Osteoporosis in a zebrafish model

**Fig. 1:**
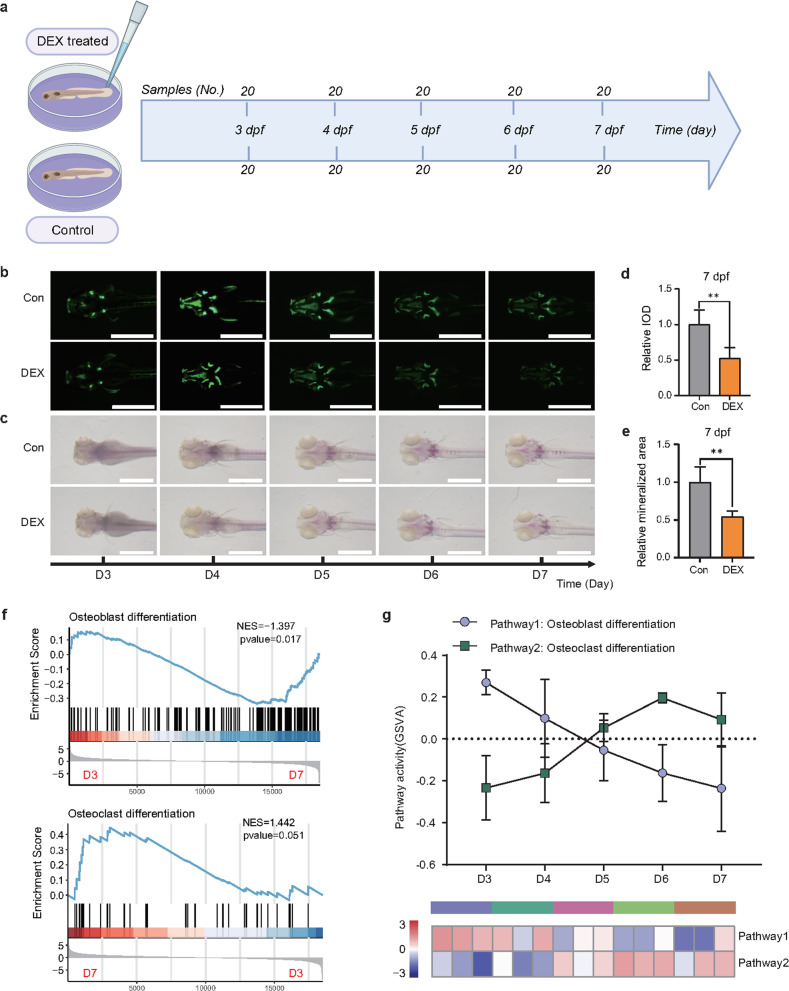
Zebrafish larva glucocorticoid-induced osteoporosis model. **a** The flowchart demonstrates the process of establishing a zebrafish osteoporosis model. Zebrafish larvae (*n* = 20) were exposed to 10 µM dexamethasone (DEX) from D3 to D7. **b** Fluorescence images in each of the osteoporotic zebrafish labeled with sp7 from D3 to D7 after the addition of DEX. Scale bars: 1 mm. **c** Alizarin red staining images in each of the osteoporotic zebrafish from D3 to D7 after the addition of DEX. Scale bars: 1 mm. **d**, **e** Semiquantitative analysis of the mineralized area and integrated optical density (IOD) at 7 days post-fertilization (dpf). **f** Gene set enrichment analysis (GSEA) of the osteoblastic (upper panel)/osteoclastic (bottom panel) differentiation signature was performed from the transcriptomes of the DEX-treated zebrafish obtained from D3 and D7. **g** The gene set variation analysis (GSVA) algorithm estimates the variation in osteoblastic/osteoclastic differentiation pathway activity over that of the DEX-treated zebrafish obtained from D3 to D7. **P < 0.01, Student’s *t*-test.

To mimic osteoporosis progression in patients, we used the zebrafish larva glucocorticoid-induced osteoporosis model, which is labeled with the stable fluorescent protein Sp7. This zebrafish model has been frequently used to discover distinct biomarkers for osteoporosis. We created the zebrafish model as shown by the flowchart in Fig. 1a. Given previous reports about this widely used DEX-induced model (Methods section) and our results from fluorescence assessment and whole-mount skeletal Alizarin red staining (ARS) histopathology (Fig. 1b–e), we demonstrated that the zebrafish larva osteoporosis model was successfully established at 7 dpf after incubation with 10 µM DEX. GSEA of differentially expressed genes (DEGs) with DEX treatment showed prominent downregulation of osteoblast differentiation and upregulation of osteoclast differentiation (Fig. 1f), suggesting the impairment of osteogenic differentiation and activation of osteoclast differentiation. GSVA revealed dynamic changes in osteoblast/osteoclast pathway activities after DEX treatment and showed that a turning point occurred on Day 6 (D6) (Fig. 1g). Collectively, these results suggested that osteoporosis emerges from dynamic cellular transition in which complex molecular networks are rewired to adapt to the disease environment.

### Dynamic gene expression during osteoporosis

**Fig. 2:**
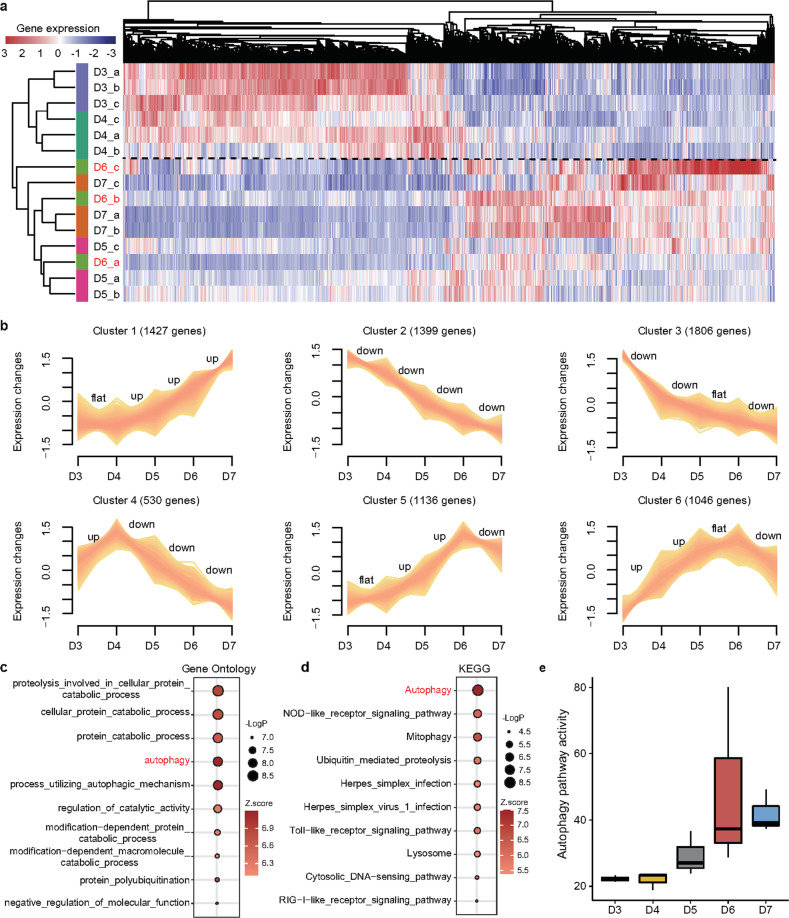
Dynamic changes in gene expression during the progression of osteoporosis. **a** The heatmap shows unsupervised hierarchical clustering analysis based on differentially expressed genes (DEGs) among the five groups. The zebrafish on D6 after the addition of DEX were not concentrated but scattered, suggesting that the zebrafish were in a special and different state from other time points. **b** The series of graphs show the dynamic changed patterns in DEGs from D3 to D7 by Mfuzz. **c**, **d** Functional enrichment of the DEGs was summarized using (**c**) annotated Gene Ontology (GO) terms and (**d**) Kyoto Encyclopedia of Genes and Genomes (KEGG) pathways. **e** Quantitative assessment of the activity of autophagy in the DEX-induced osteoporosis zebrafish.

A whole-genome expression pattern is expected to show the instant state of complex biological processes. Therefore, to comprehensively characterize the condition specificity in the progression of osteoporosis, we first established 7344 DEGs across different timepoints via diverse comparisons (false discovery rate < 0.05, Supplementary Table 4). Then, unsupervised hierarchical clustering based on these determined DEGs roughly grouped the zebrafish samples at the five time points (D3, D4, D5, D6, D7) into two independent clusters (Fig. 2a), with one mainly consisting of all samples at the third and 4th days after incubation with DEX and the other including all samples on D5, D6, and D7. This finding showed that these zebrafish from D3 to D7 exhibited advancement from a nonosteoporosis phase to an osteoporosis phase. Of note, the three samples from the 6th day did not cluster into one group (labeled in red, Fig. 2a), which indicated that zebrafish at this time point (D6) could be in the critical preosteoporosis stage.

To further illustrate the dynamic changes in gene expression levels during the progression of osteoporosis, we clustered the DEGs into six profiles (Cluster 1, …, Cluster 6) with the Mfuzz package (Fig. 2b). Cluster 1 and Cluster 2 genes were monotonically and continuously upregulated and downregulated from D3 to D6 after the addition of DEX. Cluster 3 genes were dramatically downregulated from D3 to D4 and steadily downregulated from D4 to D7, whereas genes in Cluster 4 were remarkably upregulated from D3 to D4 and dramatically downregulated from D4 to D7. Genes in Clusters 5 and 6 gradually increased at different rates and reached their highest levels on D6. These results further supported the notion that the development of osteoporosis was not gradual but instead was nonlinear with multiple drastic transitions. To determine the functional pathways that the DEGs were involved in during the development of osteoporosis, we performed GO and KEGG cascade enrichment analyses. The DEGs were strongly enriched in processes related to autophagy (Fig. 2c and d). GSVA also demonstrated the continuous activation of the autophagy pathway during the osteoporosis process (Fig. 2e). Hence, our findings were consistent with previous studies showing that the autophagic pathway was involved in the development of osteoporosis^27^.

### Identifying the triggering phase of osteoporosis by dynamic network biomarker (DNB) analysis

**Fig. 3:**
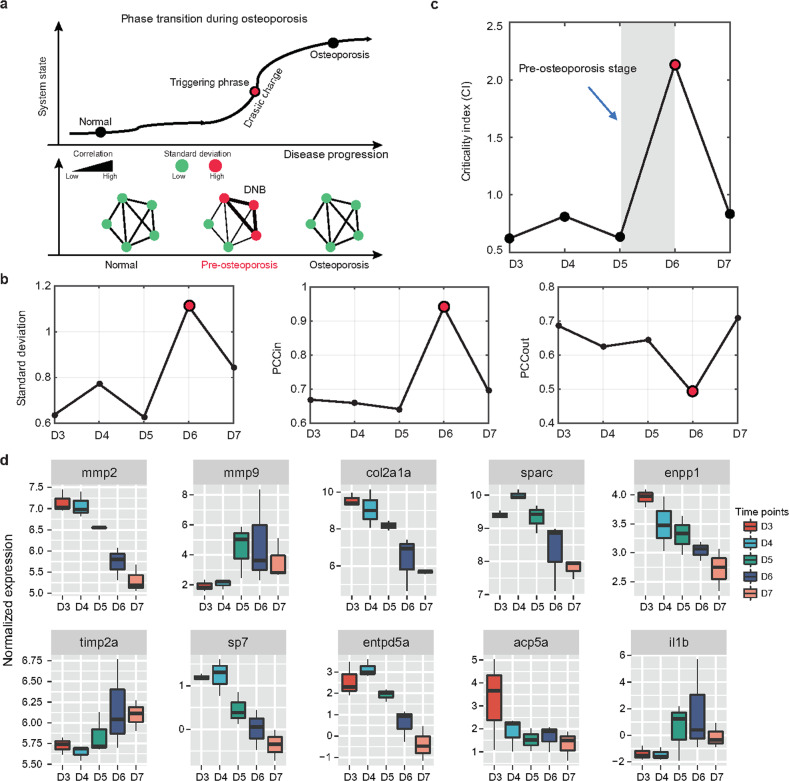
Identifying the triggering phase of osteoporosis by the DNB algorithm. **a** A schematic graph shows a phase transition during osteoporosis (upper panel). Osteoporosis progression can be divided into three stages: nonosteoporosis, preosteoporosis, and osteoporosis. Since the preosteoporosis stage is technically part of the nonosteoporosis stage, traditional biomarkers can show significant differences between the nonosteoporosis and osteoporosis stages due to their static characteristics, but they cannot be used to distinguish between the nonosteoporosis and preosteoporosis stages. Specifically, in the transition from nonosteoporosis to osteoporosis, DNB members showed an enhanced interaction with each other near the triggering phase, whereas they showed decreased interaction after the critical point (bottom panel). The diagram shows that the critical transition for osteoporosis arises at D6 after the addition of DEX, according to standard deviations, (**b**) Pearson correlation coefficients (*PCC*_*in*_ and *PCC*_*out*_) and (**c**) the criticality index over all time points in gene expression profiling. **d** Ten representative osteoporosis-associated genes reported in previous studies were selected from the DNB members and visualized.

The DNB nonlinear dynamic approach was expected to determine the triggering phase of the stage shift in the progression from nonosteoporosis to osteoporosis (Fig. 3a). We defined the triggering phase as an initial transition state from preosteoporosis to osteoporosis rather than a specific time point. The three criteria for DNB molecules are briefly summarized in Fig. 3b (Methods section). Particularly, as illustrated in Fig. 3c, from the reported time-course expression profile during the 5 days, we established the triggering phase as well as DNB members (*n* = 473) around D5 to D6 after successive exposure to DEX. Hence, the DNB score remarkably escalated when the system approached a triggering phase, suggesting a forthcoming transition in the critical state; in other words, there was a group of DNB molecules with greatly coordinated variations close to the preosteoporosis phase, in contrast with the nonosteoporosis and osteoporosis phases.

DNB molecules are considered to be functionally important biomarkers of this critical phase. We found that many known osteoporosis-associated genes reported in previous studies were identified by the DNB method (Fig. 3d), suggesting the potential power of DNBs for seeking biomarkers for osteoporosis. GO analysis of DNB genes revealed that processes related to inflammatory responses and tight junction organization were strongly enriched (Supplementary Fig. 1a). The KEGG pathway enrichment analysis also revealed the involvement of immune-associated pathways and MAPK signaling pathways in osteoporosis as functional DNB members (Supplementary Fig. 1b). Collectively, these findings suggested that the contribution of DNB genes to osteoporosis is, at least in part, due to the dysregulated expression of genes involved in the cascades required for osteoporosis.

### *cdkn1a* plays a pivotal role based on DNB ranking

**Fig. 4:**
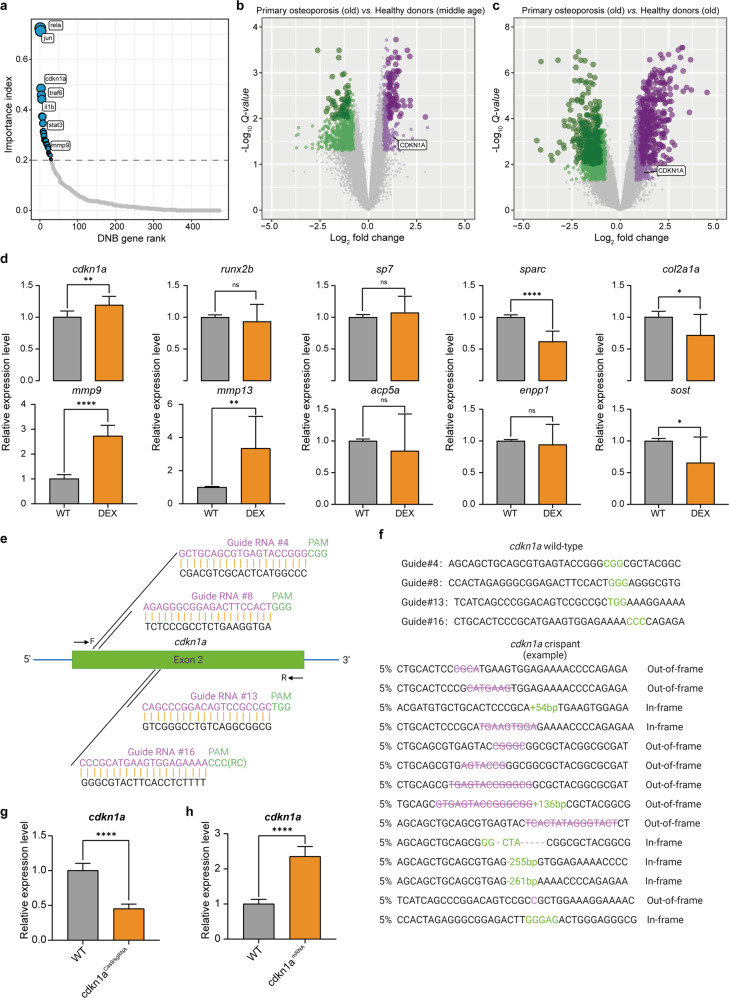
The *cdkn1a* gene is a core DNB member. **a** The diagram illustrates the DNB ranking during the critical transition of osteoporosis based on network degrees and pathway hits. **b**, **c** Volcano plot of differentially expressed genes between primary osteoporosis patients and middle-aged/elderly healthy donors. **d** The relative expression level of representative osteoporosis-associated genes was validated by quantitative real-time polymerase chain reaction (qRT‒PCR) in the wild-type (WT) and DEX-treated zebrafish (*n* = 20). **e** Diagram of the *cdkn1a*-targeting gRNA sequences. CGG, GGG, TGG and CCC (green) are the protospacer adjacent motif (PAM) sequences. Arrows indicate primer positions. **f** In *cdkn1a* crispant zebrafish, representative Sanger sequencing results of the PCR amplicons from 14 individual embryos showed different out-of-frame and in-frame indel mutations induced by Cas9/sgRNA in the targeted sites. Substitutions are represented in purple, deletions are indicated by purple strikethroughs, and insertions are highlighted in green. **g**, **h** The relative expression levels of *cdkn1a* were validated by qRT‒PCR in the WT, *cdkn1a*^Cas9/sgRNA^, and *cdkn1a*^mRNA^-treated larvae (*n* = 20). *P < 0.05, **P < 0.01, ****P < 0.0001, Student’s *t*-test.

To further investigate the functional roles of DNB genes in osteoporosis, we first ranked the DNB genes on the basis of an integrated index with two priorities, consisting of the importance in functional pathways and the significance in networks (see Methods section). Interestingly, *rela* and *jun* ranked first and second, respectively, and both have been reported to function in bone formation and osteoclast differentiation (Fig. 4a)^28–30^. However, little attention was previously paid to the function of *cdkn1a* in osteoporosis, which ranked third in our screening. In addition, differential expression analysis indicated that *CDKN1A* was remarkably upregulated in primary osteoporosis patients compared with healthy donors of both middle and old ages (Fig. 4b, c and Supplementary Fig. 2), suggesting the functional importance for osteoporosis. Furthermore, to explore the actual expression of genes on D6, we validated *cdkn1a* and several representative osteoporosis-associated genes by quantitative real-time polymerase chain reaction (qRT‒PCR) in the WT and DEX-treated zebrafish. The results showed high expression of *cdkn1a*, low expression of osteogenesis-related genes (*sp7*, *sparc*, and *col2a1a*) and high expression of osteoclast-related genes (*mmp9* and *mmp13*) in the DEX-treated group (Fig. 4d). Given these findings, we selected *cdkn1a* as the top candidate for further functional studies.

### Generation of *cdkn1a* zebrafish crispants and *cdkn1a* mutant zebrafish via the CRISPR/CAS9 system

To better understand cdkn1a function and disease mechanisms, we utilized CRISPR/Cas9-mediated gene editing to induce stable aberrations in the cdkn1a gene. We designed 16 specific sgRNAs in exon 2 of *cdkn1a* (Supplementary Table 1 and 2), and from these, we performed Sanger sequencing of cDNA and finally confirmed four efficient gRNA target sites: sgRNA4, sgRNA8, sgRNA13, and sgRNA16 (Fig. 4e and Supplementary Fig. 3). With this technique, the pool of sgRNAs was coinjected with Cas9 protein in the one-cell stage of the zebrafish embryos, generating F0 *cdkn1a*^Cas9/sgRNA^ mosaic knockout embryos, hereafter referred to as “crispants”^31^. It is currently not possible to verify the protein levels of Cdkn1a in the crispants by western blots due to the lack of a relevant and reliable antibody. Instead, the PCR products were cloned into T vectors for direct DNA sequencing. A total of 20 individual crispant embryos showed 70% insertion or deletion (indel) mutations when injected with sgRNA4, sgRNA8, sgRNA13, and sgRNA16 (Fig. 4f). Additionally, qRT‒PCR showed a notable decrease in *cdkn1a* crispant transcript abundance (Fig. 4g). These results have proven the successful generation of *cdkn1a* knockout crispants. In addition, *cdkn1a*-overexpressing embryos were constructed accordingly. The qRT‒PCR results showed a notable increase in cdkn1a expression levels in the *cdkn1a*^mRNA^-treated group versus the WT group (Fig. 4h).

Furthermore, the siblings of these microinjected F0 embryos were raised to adulthood and outcrossed with WT fish to produce F1 embryos (Supplementary Fig. 4c). Two heritable mutations were obtained, namely, a −26 bp deletion and a −3-6 bp deletion, and F1 fish containing the same mutation were crossed to obtain homozygous *cdkn1a* mutant lines. The sequencing results are shown in Supplementary Fig. 4d and 4e.

### *cdkn1a* participates in osteoclast differentiation by regulating its upstream regulators

**Fig. 5:**
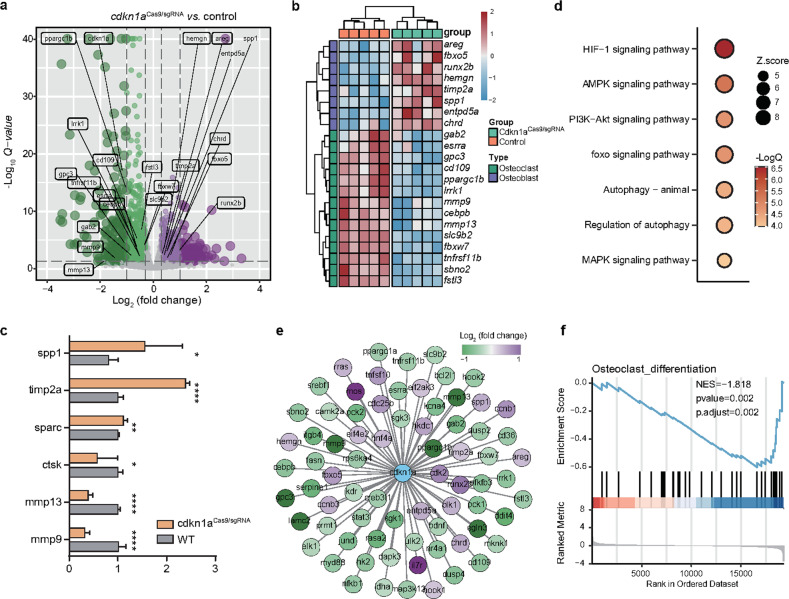
A molecular network required for osteoclast differentiation is modulated by *cdkn1a*. **a** Differentially expressed genes (|log2Fold change | > 0.3 and FDR < 0.05 were used as thresholds) after *cdkn1a* knockout (*n* = 2437 DEGs). **b** Heatmap of the osteoblast- and osteoclast-associated genes between the *cdkn1a*^Case9/sgRNA^ and control groups. **c** The expression changes of representative DEGs after *cdkn1a* knockout were validated by qRT‒PCR. **d** Functional enrichment analysis showing significant KEGG pathways among *cdkn1a*-regulated genes. **e** Global view of the *cdkn1a-*mediated molecular network required for osteoporosis. **f** GSEA showed that osteoclast differentiation was negatively enriched using *cdkn1a*-regulated genes.

To determine how *cdkn1a* affects functional pathways, we performed RNA-seq on wild-type and *cdkn1a* knockout zebrafish. We found 2437 DEGs upon *cdkn1a* knockout, 1462 downregulated and 975 upregulated DEGs (Fig. 5a). Notably, a few genes associated with osteogenesis, such as *spp1, timp2a*, and *sparc*, were activated, whereas those associated with osteoclast differentiation, such as *ctsk, mmp13*, and *mmp9*, were repressed upon *cdkn1a* knockout (Fig. 5b). These data were confirmed by qRT‒PCR (Fig. 5c). Functional enrichment analysis suggested that *cdkn1a*-regulated genes were mainly related to HIF-1^32^, AMPK^33^, PI3K-AKT^34^, FoxO^35^, autophagy^36^, and MAPK signaling cascades (Fig. 5d)^37^, all of which have been demonstrated to be upstream regulators of osteoclast proliferation or differentiation. These findings suggested that *cdkn1a* is connected to a set of functional molecules required for osteoclastogenesis (Fig. 5e). To find evidence supporting this, we performed GSEA, and the results clearly indicated a strong decrease in osteoclast differentiation processes upon *cdkn1a* knockout (Fig. 5f). The transcriptome data supported the functional role of *cdkn1a* in osteoclast differentiation.

### *cdkn1a* negatively regulates osteogenesis of zebrafish larvae

**Fig. 6:**
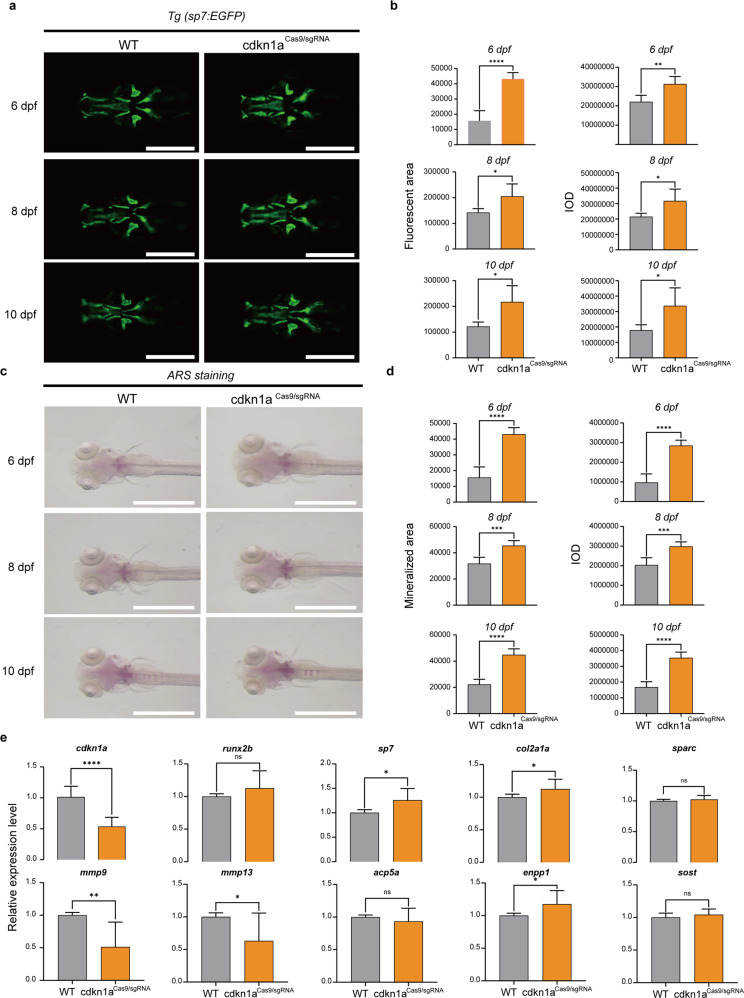
*cdkn1a* crispant zebrafish larvae show increased bone mineralization. **a** Green fluorescent protein (GFP) images of WT and *cdkn1a*^Cas9/sgRNA^-treated zebrafish head regions at 6, 8, and 10 dpf. Scale bars: 500 μm. **b** Semiquantitative analysis of GFP area and IOD (*n* = 6). **c** Alizarin red staining evaluated the effect of *cdkn1a*^Cas9/sgRNA^ treatment on bone mineralization at 6, 8, and 10 dpf compared to that in the WT group. Scale bars: 1 mm. **d** Semiquantitative analysis of Alizarin red staining activity (*n* = 6). **e** mRNA expression levels of genes related to osteoblasts (*runx2b, sp7, col2a1a, sparc*) and osteoclasts (*mmp9, mmp13, acp5a, enpp1, sost*) in the WT and *cdkn1a*^Cas9/sgRNA^-treated groups (*n* = 20). *P < 0.05, **P < 0.01, ***P < 0.001, ****P < 0.0001, Student’s *t*-test.

**Fig. 7:**
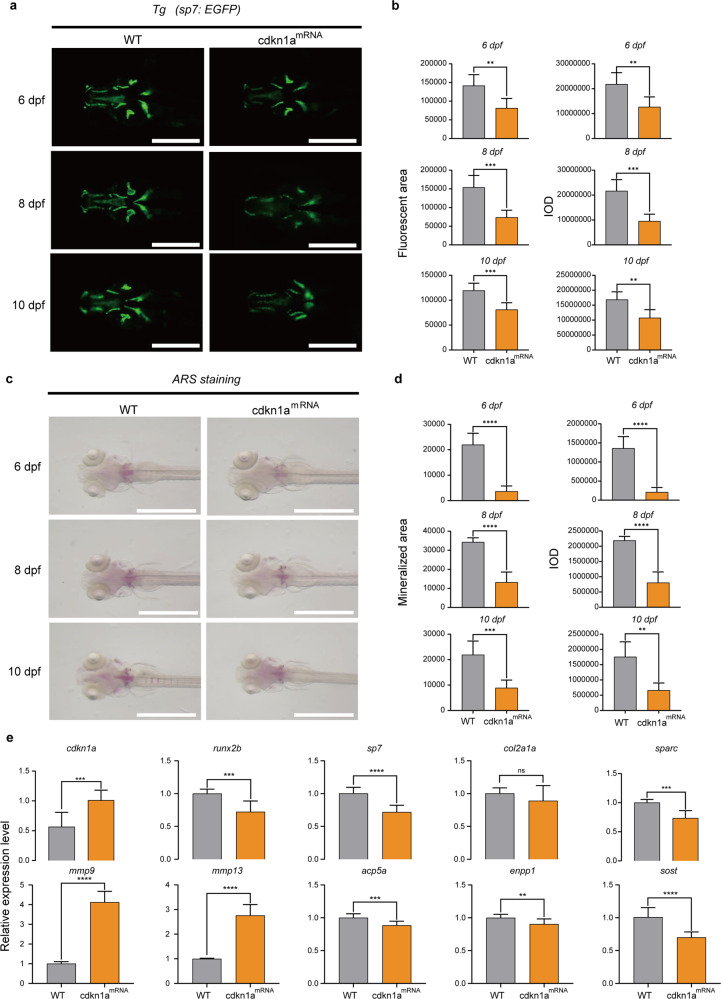
*cdkn1a*^mRNA^-treated zebrafish larvae showed decreased bone mineralization. **a** GFP images of WT and *cdkn1a*^mRNA^-treated zebrafish head regions at 6, 8, and 10 dpf. Scale bars: 500 μm. **b** Semiquantitative analysis of GFP area and IOD (*n* = 6). **c** Alizarin red staining evaluated the effect of *cdkn1a*^mRNA^ treatment on bone mineralization at 6, 8, and 10 dpf compared to that of the WT group. Scale bars: 1 mm. **d** Semiquantitative analysis of Alizarin red staining activity (*n* = 6). **e** mRNA expression levels of genes related to osteoblasts (*runx2b, sp7, col2a1a, sparc*) and osteoclasts (*mmp9, mmp13, acp5a, enpp1, sost*) in the WT and *cdkn1a*^mRNA^-treated groups (*n* = 20). *P < 0.05, **P < 0.01, ***P < 0.001, ****P < 0.0001, Student’s *t-*test.

To determine the effect of *cdkn1a* on the formation of early skeletal elements, we performed loss-of-function and gain-of-function experiments and visualized mineralized bone by expressing green fluorescent proteins and ARS in Tg (*Ola. Sp7: nlsGFP*) zebrafish larvae. Bone mass and density were calculated by measuring the fluorescent area and IOD in the WT-, *cdkn1a*^Cas9/sgRNA^, and *cdkn1a*^mRNA^-treated groups at 6, 8, and 10 dpf (Fig. 6a and Fig. 7a). The results of the quantitative analysis showed that the green fluorescence area and IOD of head bone in Tg (*Ola. Sp7: nlsGFP*) zebrafish larvae were significantly higher in *cdkn1a*^Cas9/sgRNA^ but were lower in *cdkn1a*^mRNA^ compared with WT (Fig. 6b and Fig. 7b). Moreover, ARS (Fig. 6c and Fig. 7c) results demonstrated that *cdkn1a*^Cas9/sgRNA^ obviously promoted calcium deposition, whereas *cdkn1a*^mRNA^ impaired the process, leading to decreased mineralization of the notochord. Furthermore, semiquantitative analysis of ARS activity revealed a similar pattern (Fig. 6d and Fig. 7d).

To further explore the regulatory mechanism of *cdkn1a*, we performed gene expression analysis. Based on previous research, we selected several osteoblast-related genes (*runx2b, sp7, col2a1a, sparc*) and several osteoclast-related genes (*mmp9, mmp13, acp5a, enpp1, sost*). The mRNA expression levels of the osteoblast-related genes *sp7* and *col2a1a* were significantly higher than those in the WT group, and the osteoclast-related genes *mmp9* and *mmp13* were strongly downregulated in the *cdkn1a*^Cas9/sgRNA^ group (Fig. 6e). In contrast, overexpression of *cdkn1a* in zebrafish markedly suppressed the osteoblast-related genes *runx2b, sp7*, and *sparc* and significantly upregulated the osteoclast-related genes *mmp9* and *mmp13* (Fig. 7e). Furthermore, *enppl* was enhanced in the *cdkn1a*^Cas9/sgRNA^ group, and *acp5a, enppl*, and *sost* were markedly suppressed in the *cdkn1a*^mRNA^ group compared to the WT group (Fig. 6e and Fig. 7e).

To determine the knockout effect of *cdkn1a* on bone formation and gene expression, we performed ARS and qRT‒PCR in the WT and *cdkn1a* mutant groups. The results of the quantitative analysis showed that the mineralized area and IOD were significantly higher in the *cdkn1a* mutant group at 6, 7, 8, 9, and 10 dpf than in the WT group (Supplementary Fig. 5a and 5b). Furthermore, the mRNA expression levels of most osteoblast-related genes were significantly increased in the *cdkn1a* mutant group compared with the WT group. Although the mRNA expression levels of the osteoclast-related genes *mmp9, enpp1, ctsk*, and *rankl* showed a slight increase in the *cdkn1a* mutant groups, statistical significance was not reached (Supplementary Fig. 5c). Collectively, these findings again proved that *cdkn1a* negatively regulates the osteogenesis of zebrafish larvae.

### The *cdkn1a* mutant rescued osteogenesis of zebrafish larvae

**Fig. 8:**
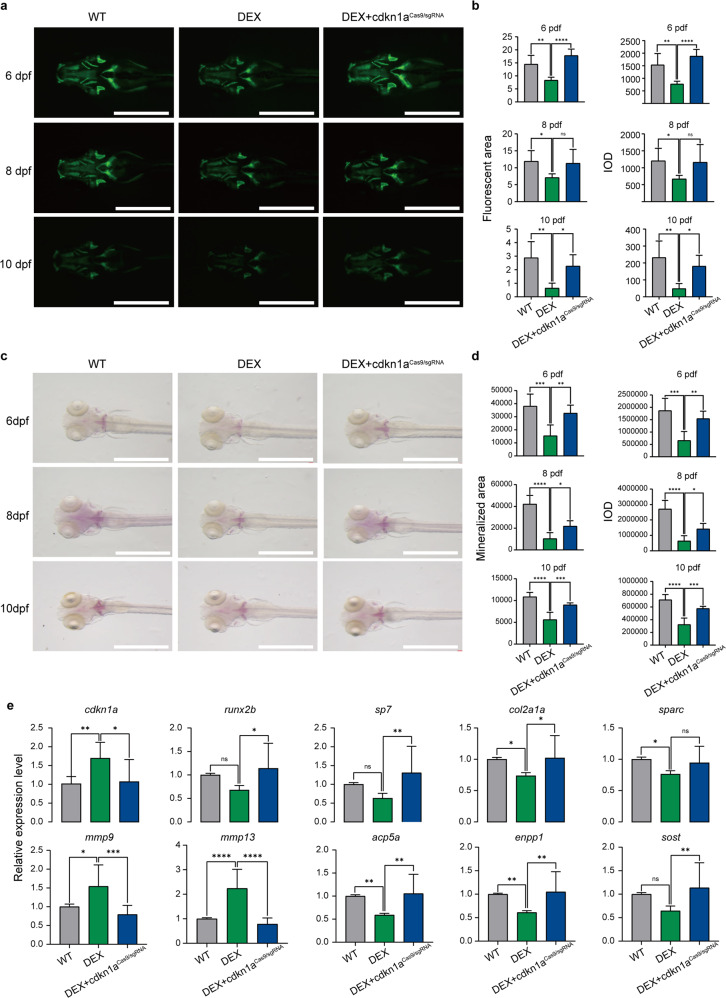
The cdkn1a mutant rescued the osteogenesis of zebrafish larvae. **a** GFP images of WT, DEX-treated and DEX-treated *cdkn1a*^Cas9/sgRNA^ zebrafish head regions at 6, 8, and 10 dpf. Scale bars: 1 mm. **b** Semiquantitative analysis of GFP area and IOD (*n* = 6). **c** Alizarin red staining was used to evaluate the bone mineralization in the WT, DEX-treated, and DEX-treated *cdkn1a*^Cas9/sgRNA^ groups at 6, 8, and 10 dpf. Scale bar: 1 mm. **d** Semiquantitative analysis of Alizarin red staining activity (*n* = 6). **e** The mRNA expression levels of genes related to osteoblasts (*runx2b, sp7, col2a1a, sparc*) and osteoclasts (*mmp9, mmp13, acp5a, enpp1, sost*) in the WT, DEX-treated, and DEX-treated *cdkn1a*^Cas9/sgRNA^ groups (*n* = 20). *P < 0.05, **P < 0.01, ***P < 0.001, ****P < 0.0001, one-way ANOVA.

Previous investigations have documented that DEX remarkably reduces bone mineralization in zebrafish, suggesting that DEX is involved in modulating skeletal development. Whether *cdkn1a* participates in this process remains unclear. To explore the restorative effects of *cdkn1a* knockdown on the osteoporosis model, we performed *cdkn1a* knockdown on zebrafish at 6, 8, and 10 dpf while adding 10 μM DEX mix. Morphological analysis of fluorescent images showed that the DEX-treated group exhibited a significant reduction in fluorescent area and IOD at 6, 8, and 10 dpf in contrast to the WT group, but the DEX-treated *cdkn1a*^Cas9/sgRNA^ group had a greater fluorescent area and IOD than the DEX-treated group, comparable to the WT group (Fig. 8a, b). This finding suggested that *cdkn1a* knockout remarkably inhibits bone loss induced by DEX treatment. These results were consistent with the image analysis of the conventional ARS group (Fig. 8c, d). For gene expression analysis, zebrafish larvae treated with DEX showed a trend toward a reduction in the expression of osteoblast-related genes (*runx2b, sp7, col2a1a*, and *sparc*) and an increase in the expression of osteoclast-related genes *(mmp9* and *mmp13*) compared with those of the corresponding control group (Fig. 8e). Importantly, *cdkn1a*^Cas9/sgRNA^ inhibited the downregulation of osteoblast-specific genes and the upregulation of osteoclast-specific genes induced by DEX. The above results suggested that knockout stimulation of *cdkn1a* can rescue the bone loss caused by DEX.

## Discussion

Given the high prevalence and seriousness of osteoporosis—a persistent public health issue manifesting as reduced bone strength coupled with an elevated risk of fractures—understanding its pathogenic mechanisms is urgent. One major problem with the treatment strategy of osteoporosis is the inability to identify its triggering phase to enable early precise intervention. To address this, we developed a model for predicting osteoporosis in zebrafish based on the DNB algorithm^7,8^. In contrast with traditional molecular signatures that determine disease condition by comparing the expression of biomolecules in a static approach, the DNB method was superior in establishing the predisease phase (a triggering phase prior to the drastic transition to the disease phase) during the progress of disease through the crosstalk of biomolecules (differential networks) via a dynamic approach.

Here, we established a zebrafish osteoporosis model and evaluated the time-dependent changes in sp7 fluorescence and IOD of ARS upon the addition of DEX from D3 to D7, where a gradual decrease in the intensity was observed. In contrast to molecular signatures identified empirically via “omics” to be “differentially expressed,” DNB theory identified the 6th day as the key node between the healthy state and osteoporosis for the first time, moving the triggering phase prior to the drastic transition to the osteoporosis phase forward by 2 days (from 8 dpf to 6 dpf)^13,38^, and it identified *cdkn1a* as a core member of DNBs. The zebrafish gene *cdkn1a* is a cyclin-dependent kinase (Cdk) inhibitor protein and p53 target gene located on chromosome 22, and it negatively regulates the cell cycle process and prevents DNA replication^39^. It is a key sentinel of the cell cycle and plays an important role in a variety of cellular and physiological processes, including cell proliferation, differentiation, and apoptosis^40^. Regarding the role of *cdkn1a* in bone formation, there are different points of view. Some scholars have reported that the inhibition of *cdkn1a* promotes osteogenic differentiation in progenitor/stem cells^41^. Others have reported that *cdkn1a* can resist osteoporosis by activating the Nrf2/HO-1 signaling pathway^42^. However, the role of *cdkn1a* in zebrafish osteoporosis initiation remains unclear.

The loss and gain of function of osteogenic or osteoclast-related genes are closely associated with the development of osteoporosis. In recent years, RNA-guided CRISPR‒Cas9, a novel genetic approach, has provided an effective means to introduce reusable targeted loss-of-function mutations at specific loci in the genome^43^. To further explore the role of genes involved in osteoporosis, we established one-step generation of an efficient F0 mosaic knockout (crispant) model by CRISPR/Cas9-mediated gene editing with multiple sgRNAs used in zebrafish embryos in this study; this approach was proven by previous studies to have the potential to approximate the stable mutant genotype, so its off-target efficiency could be ignored^31,44^. Based on the above evidence, we further elucidated the function of *cdkn1a* and the disease mechanisms involved in the onset of osteoporosis. Additionally, to further test our hypothesis, we established a pure knockout line. The *cdkn1a* knockout effect indeed confirmed the robustness of our findings about the role of *cdkn1a* and usefulness of the marker as the triggering event in the progression of osteoporosis.

Differential expression analysis between WT and mosaic knockout larvae revealed the underlying mechanisms of *cdkn1a* in the preosteoporosis phase at the network level. Consistent with previous findings, our results suggested that the genes regulated by *cdkn1a* mainly participated in the upstream regulators of osteoclast differentiation. For instance, bone resorption can be stimulated in a HIF-1-dependent manner^45^, and AMPK activation may promote the progression of osteoclastic differentiation^46^. In addition, sustained phosphoinositide-3-kinase (PI3K) signaling elevates osteoclastogenic potential^47^. These results demonstrated that *cdkn1a* was involved in the pathogenesis of osteoporosis, at least in part, by regulating osteoclast differentiation.

To better illustrate the role of *cdkn1a* as an osteoporosis promotor, we performed bone mineralization and endogenous genetic tests on *cdkn1a* knockout and overexpression models. The former was conducted by CRISPR‒Cas9 as previously mentioned, and the latter was generated by the pXT-7 vector. We found that *cdkn1a* repressed bone mineralization and that the expression changes in osteoclast-related genes were more significant than those in osteoblast-related genes. In addition, we tested whether the effect of an osteoporosis inducer, such as DEX, could be reversed by *cdkn1a* knockout. We found that *cdkn1a* knockout reversed the decreased bone mass induced by DEX. Moreover, the alteration of osteoblast-related genes and osteoclast-related gene degradation can be reversed. These results indicate that *cdkn1a* knockout can overcome the osteoporosis phenotype and molecular changes induced by DEX treatment. Overall, our findings confirmed the reliability of the DNB method and the key function of *cdkn1a* in osteoporosis.

Our findings also increased our interest in the relationship between osteoporosis and the expression of the senescence marker *cdkn1a*^48^. In the *cdkn1a* gain-of-function zebrafish model, osteoclast-related gene expression was upregulated, accompanied by mineral absorption, but osteoblast-related gene expression was not changed significantly, which demonstrated a relationship between age and the presence of drug-induced osteoporosis. Previous studies have reported similar results on relationships between aging and osteoporosis^49,50^. These interesting results hinted at the possibility of developing an overlapping strategy for the treatment of aging and osteoporosis or age-related skeletal aging, which would be quite distinct from the current pharmacotherapy for osteoporosis. For example, clearance of senescent cells could slow bone loss, whether age-related or drug-induced, and prevent or treat osteopenia and osteoporosis. Furthermore, another pathway to the development of osteoporosis could be drug-induced disturbance of bone mass leading to phenotypic features suggestive of aging; this may be evidence of a novel aging phenotype associated with bone loss. There is often a reduced bone mass during aging, and whether bone loss could be a hallmark of aging deserves further investigation. Thus, determining the importance of controlling bone mass in the context of aging will further broaden the therapeutic arsenal. Although this study revealed essential discoveries, there are some limitations. First, the current zebrafish model of osteoporosis is mainly induced by glucocorticoids, and its pathological mechanism may not be identical to that of primary osteoporosis. Second, zebrafish larvae are relatively small, so some assays are difficult to perform, such as serum metabolic correlation studies, indicating that more research is needed on larger mammals. Overall, these could be fruitful areas to explore in further research.

In conclusion, we established for the first time a novel way to determine the triggering phase of osteoporosis on the 6th day after DEX treatment, and we provide biological insights into the molecular pathogenesis of osteoporosis from the perspectives of dynamics and networks. In this study, a crispant zebrafish model and a knockout zebrafish model were successfully generated by CRISPR/Cas9 technology. Based on these models, we found that *cdkn1a* influences skeletal development and bone mineralization. Mutations in *cdkn1a* altered the expression of osteoblast-related and osteoclast-related genes. Further experiments revealed that *cdkn1a*-regulated genes were mainly involved in upstream regulators of osteoclast proliferation or differentiation. This study provides a basis to determine the role of *cdkn1a* in bone metabolism and development. We proposed *cdkn1a* as an early-signaling indicator of the preosteoporosis state, against which therapies could be designed, thereby improving our understanding of the molecular pathogenesis of osteoporosis onset for early diagnosis and even mitigation.

## Supplementary information

Supplementary information

Supplementary Table S4

Supplementary Table S5

## Data Availability

All data needed to evaluate the conclusions in the paper are presented in the paper and the Supplementary Information. The GEO accession number for the data reported in this paper is GSE194301.
