# Supplementary material for: Dynamic network biomarker identifies cdkn1a-mediated bone mineralization in the triggering phase of osteoporosis
Source: Exp Mol Med. 2023 Jan 4;55(1):81–94. doi: 10.1038/s12276-022-00915-9 (PMC9898265; doi:10.1038/s12276-022-00915-9)
Supplement: Supplementary file 1 — Supplementary information [file 12276_2022_915_MOESM1_ESM.pdf]

Supplementary information

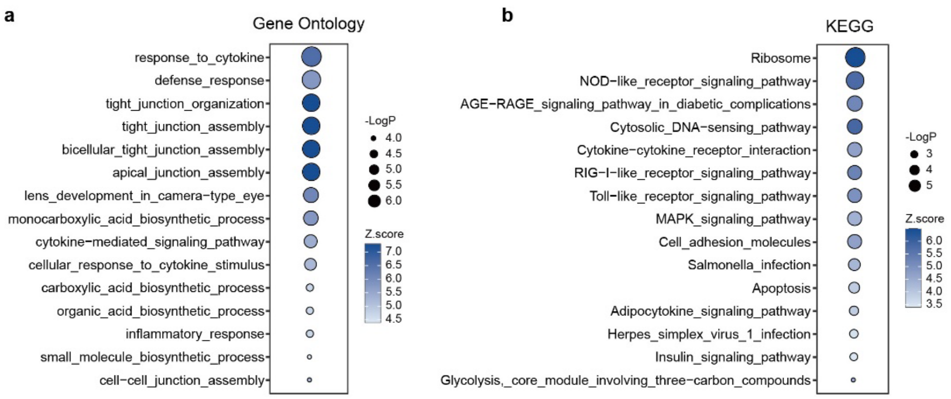

Supplementary Fig. 1. Gene ontology (GO) and pathway enrichment analysis of DNB genes. (a) GO terms enriched in the differentially expressed genes (DEGs). (b) Kyoto Encyclopedia of Genes and Genomes (KEGG) pathways enriched in the DEGs.

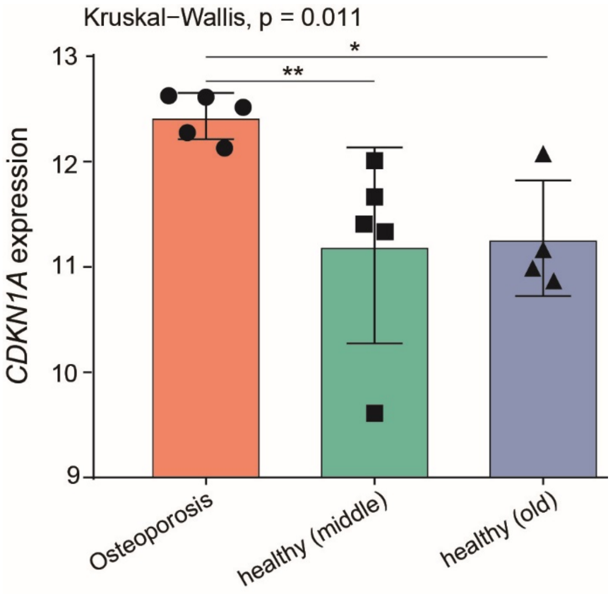

Supplementary Fig. 2. Differentially expression analysis indicated that *CDKN1A* was remarkably upregulated in primary osteoporosis patients in contrast with healthy donors with both middle and old ages.

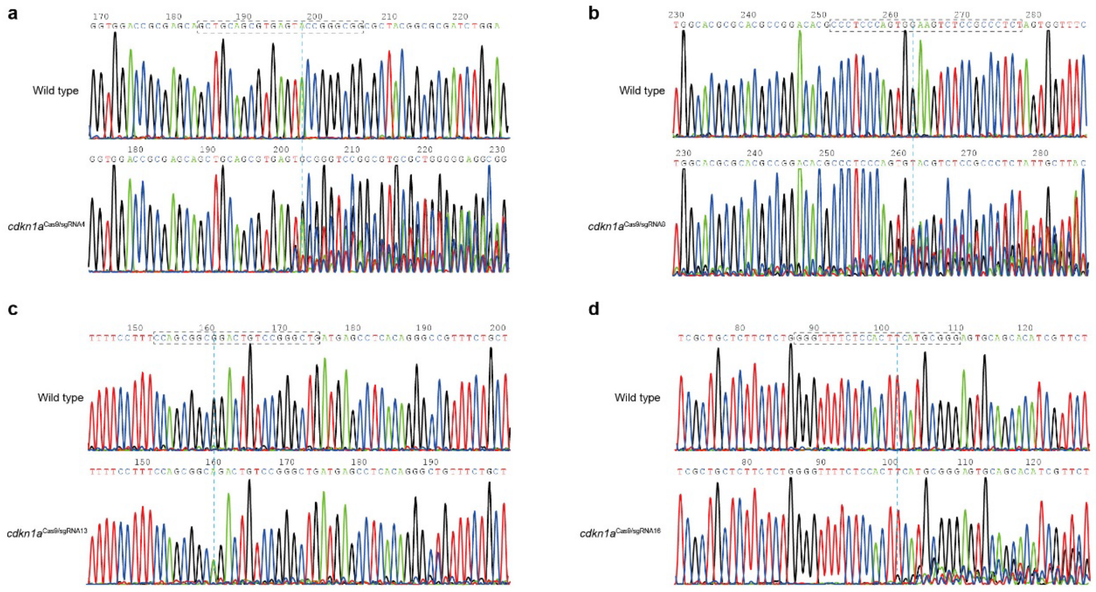

Supplementary Fig. 3. Mutation of the target sites with the gRNAs was confirmed by Sanger sequencing. (a) Sequencing peaks for wild type (WT) and *cdkn1a*$^{Cas9/sgRNA4}$ crispants. (b) Sequencing peaks for WT and *cdkn1a*$^{Cas9/sgRNA8}$ crispants. (c) Sequencing peaks for WT and *cdkn1a*$^{Cas9/sgRNA13}$ crispants. (d) Sequencing peaks for WT and *cdkn1a*$^{Cas9/sgRNA16}$ crispants. The black dotted box marks the target sequence. The blue dotted line marks the start position of mutation.

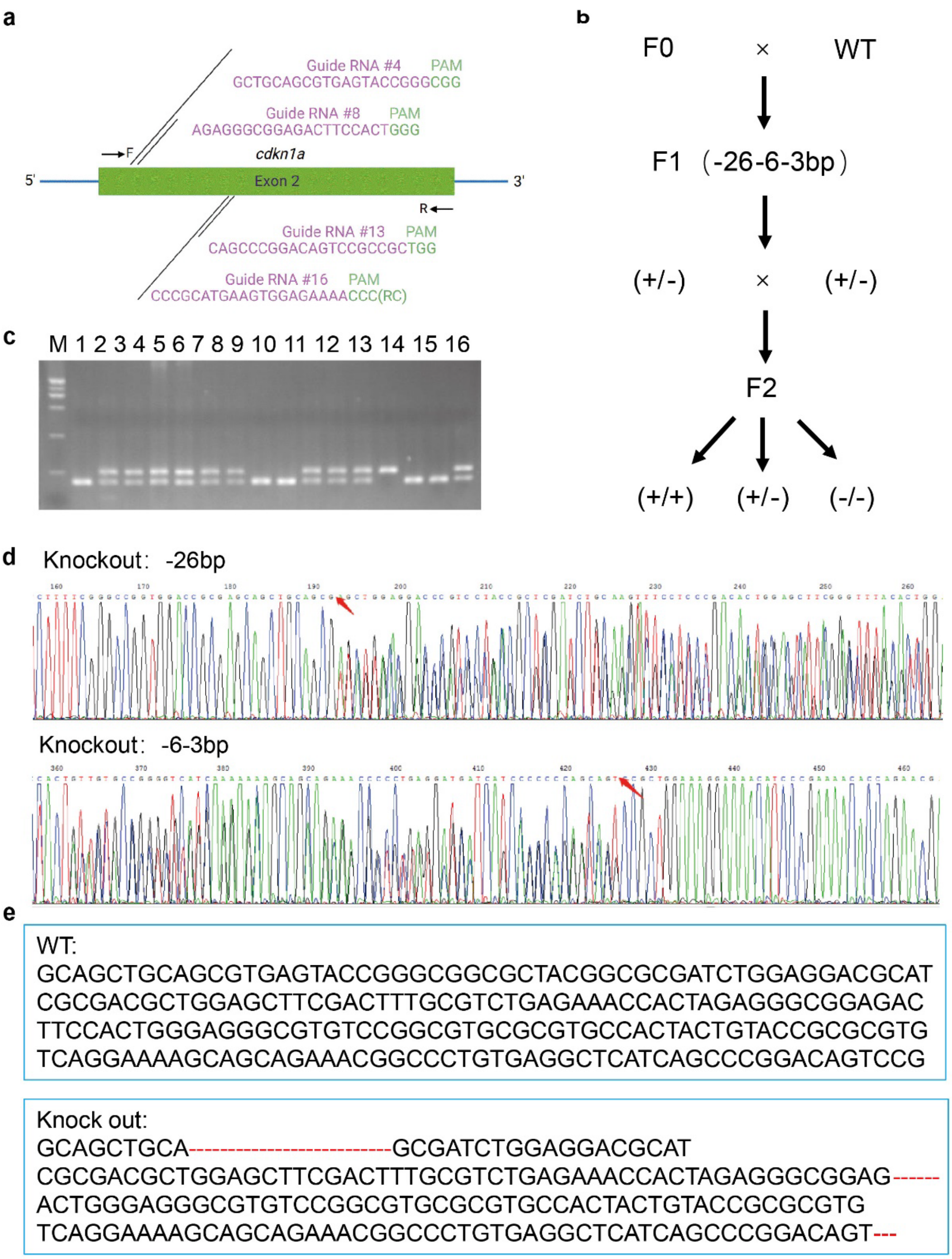

Supplementary Fig. 4. Establishment of a homozygous knockout model of the cdkn1a gene in zebrafish. (a) The *cdkn1a* sgRNAs site in exon-2. (b) Diagram detailing crossing scheme used to obtain a homozygous strain. (c) Gel electrophoresis of PCR products of F1 mutant. (d) The F1 mutant sequencing result. The red arrow indicates the starting point of the frameshift mutation. (e) Sequence alignment result. The red strikethroughs indicate frameshift mutations.

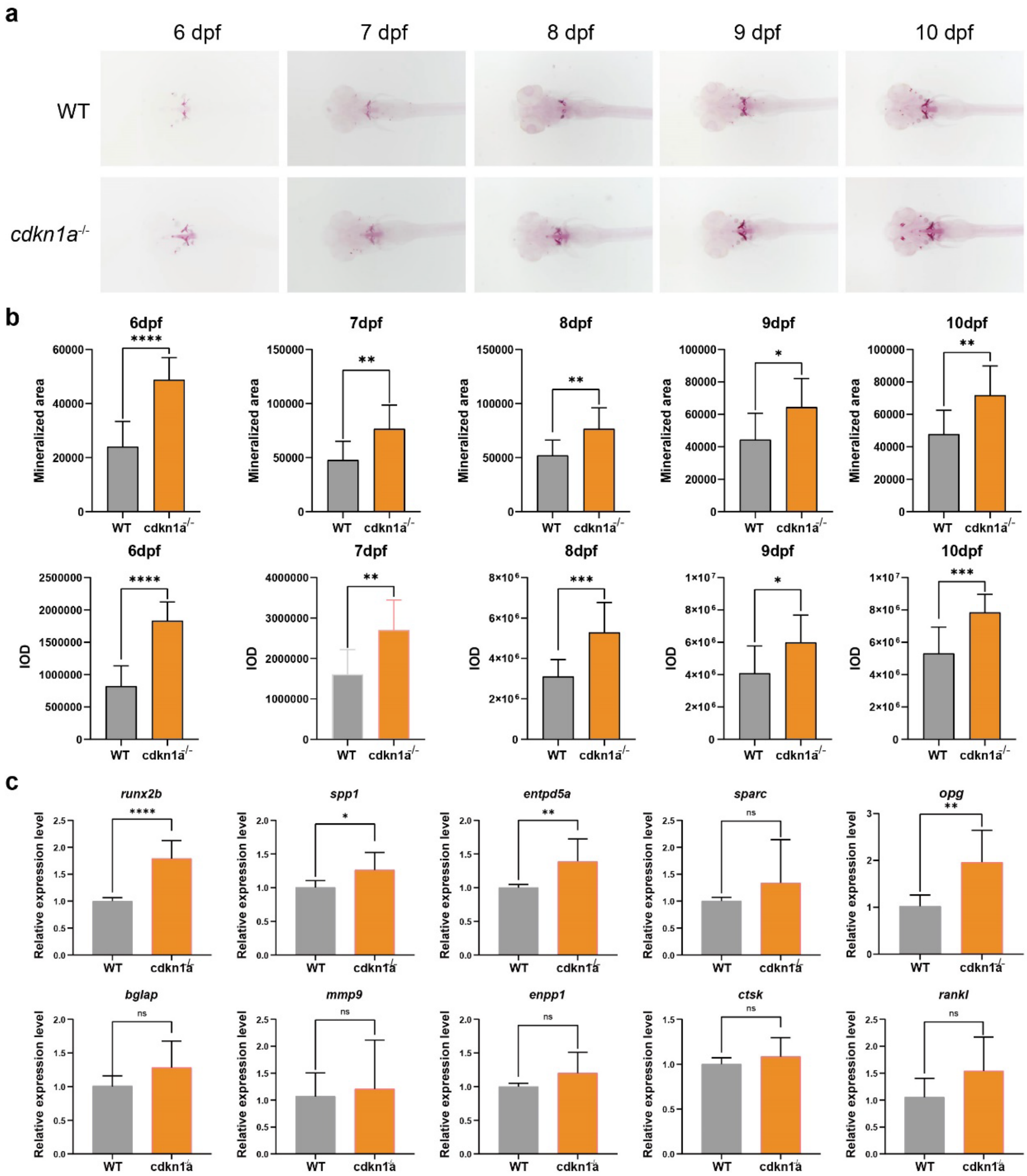

Supplementary Fig. 5. The *cdkn1a*-mutant zebrafish larvae show increased bone mineralization. (a) Alizarin red staining evaluated the *cdkn1a*-mutant group on the bone mineralization at 6, 7, 8, 9, and 10 days post-fertilization (dpf) compared to the WT group. Scale bars: 1 mm. (b) Semiquantitative analysis of mineralization area and IOD (n = 10). (c) mRNA expression levels of genes related to osteoblasts (*runx2b, spp1, endp5a, sparc, bglap*) and osteoclasts (*mmp9, enpp1, ctsk, rankl, opg*) in WT and *cdkn1a*-mutant group (n = 20). *P < 0.05, **P < 0.01, ***P < 0.001, ****P < 0.0001, Student's *t*-test.

Supplementary Table 1. Exon 2 sequence of *cdkn1a*.

ATGGCGGCGCACAAGCGGATCCTACGTTCACTCGGTAATGGGCCGACTAGGCGGA<br>GTCTTTTCGGGCCGGTGGACCGCGAGCAGCTGCAGCGTGAGTACCGGGCGGCGCTA<br>CGGCGCGATCTGGAGGACGCATCGCGACGCTGGAGCTTCGACTTTGCGTCTGAGAA<br>ACCACTAGAGGGCGGAGACTTCCACTGGGAGGGCGTGTCCGGCGTGCGCGTGCCA<br>CTACTGTACCGCGCGTGTCAGGAAAAGCAGCAGAAACGGCCCTGTGAGGCTCATC<br>AGCCCGGACAGTCCGCCGCTGGAAAGGAAAACATCCCGAAAACACCAGAACGATG<br>TGCTGCACTCCCGCATGAAGTGGAGAAAACCCCAGAGAAGAGCAGCGAGCTGAAG<br>CGCAAACAGACCAACATCACAG

Supplementary Table 2. ***cdkn1a*** **RNA guides for CRISPR.**

Guides	Target sequence	Reverse complement sequence
Guide 1	ATCCTACGTTCACTCGGTAATGG	CCATTACCGAGTGAACGTAGGAT
Guide 2	CGACTAGGCGGAGTCTTTTCGGG	CCCGAAAAGACTCCGCCTAGTCG
Guide 3	TGCAGCTGCTCGCGGTCCACCGG	CCGGTGGACCGCGAGCAGCTGCA
Guide 4	GCTGCAGCGTGAGTACCGGGCGG	CCGCCCGGTACTCACGCTGCAGC
Guide 5	GGCGGCGCTACGGCGCGATCTGG	CCGCCGCGATGCCGCGCTAGACC
Guide 6	TGGAGGACGCATCGCGACGCTGG	CCAGCGTCGCGATGCGTCCTCCA
Guide 7	TGCGTCTGAGAAACCACTAGAGG	CCTCTAGTGGTTTCTCAGACGCA
Guide 8	AGAGGGCGGAGACTTCCACTGGG	CCCAGTGGAAGTCTCCGCCCTCT
Guide 9	CAGTAGTGGCACGCGCACGCCGG	CCGGCGTGCGCGTGCCACTACTG
Guide 10	ACTACTGTACCGCGCGTGTCAGG	CCTGACACGCGCGGTACAGTAGT
Guide 11	GCAGCAGAAACGGCCCTGTGAGG	CCTCACAGGGCCGTTTCTGCTGC
Guide 12	TCCGGGCTGATGAGCCTCACAGG	CCTGTGAGGCTCATCAGCCCGGA
Guide 13	CAGCCCGGACAGTCCGCCGCTGG	CCAGCGGCGGACTGTCCGGGCTG
Guide 14	GATGTTTTCCTTTCCAGCGGCGG	CCGCCGCTGGAAAGGAAAACATC
Guide 15	CACATCGTTCTGGTGTTTTCGGG	CCCGAAAACACCAGAACGATGTG
Guide 16	GGGTTTTCTCCACTTCATGCGGG	CCCGCATGAAGTGGAGAAAACCC

Supplementary Table 3. Primer sequences for the genes observed in qRT-PCR.

Gene	Forward Primer (5'-3')	Reverse Primer (5'-3')
β-actin	ACGAACGACCAACCTAAACTCT	TTAGACAACTACCTCCCTTTGC
cdkn1a	CGCAAACAGACCAACATCAC	CGGAATAAACGGTGTCGTCT
runx2b	GAAGCGGAAGAGGGAAGAGC	AATGGCTCTGTGGTAAGTGGC
sp7	TCCTCTCCCGCTTTTGGATT	TCCCGAATTTGTTGCAGGTC
col2a1a	GATGTGGAGATCAGAGCAGAGGG	CATGATGGGCAGTCTGGATGTCT
sparc	CCCTGCAAATACATCGCTCC	TCTCGTAAATCTTCTTCACCCTG
mmp9	CAGAGAGGAAAAGGCAAGGTG	AGGAATACATCATGTGAATCAATG
mmp13	AGACCAGGACACACTCGCAGAG	CTGCTGCATCTCCTTGAGCCT
acp5a	CCCCATAGAGACCGCTACAG	ACATCAGTGACGCCCTTGTA
enpp1	TTGCAACCAACTCAGCTCTG	CCCACTTTCACACAGTCTGC
sost	TACCAGAATACGCGGAGGAC	AAGTCCGTGTGTTTGCTGAC
entpd5a	CGCTACCGTGCAATCAATCA	AAGACGCGACTGAAGTTGAC
bglap	TGAAGAGCCTGACAGTCCTG	CACGCTTCACAAACACACCT
ctsk	CTATAAAGAGATTCCTCAGGG	ACACGGGTCCCACATTGG
rankl	CTCACCTTCCAATCAAGACGCCC	CTTTCATGCCATCCCAGGCTATCT
opg	GTCAAAACCGCTGGAACGCC	CAGCAGATGCTCTTCCCCCTG

Supplementary Table 4. (separate file)

7344 differentially expressed genes (DEGs).

Supplementary Table 5. (separate file)

The total genes of “osteoblast differentiation” and “osteoclast differentiation”.
